# Scratchpads 2.0: a Virtual Research Environment supporting scholarly collaboration, communication and data publication in biodiversity science

**DOI:** 10.3897/zookeys.150.2193

**Published:** 2011-11-28

**Authors:** Vincent S. Smith, Simon D. Rycroft, Irina Brake, Ben Scott, Edward Baker, Laurence Livermore, Vladimir Blagoderov, David Roberts

**Affiliations:** 1 Natural History Museum, Cromwell Road, London, SW7 5BD, U.K.

**Keywords:** Taxonomy, database, Virtual Research Environment, Biodiversity, e-infrastructure

## Abstract

The Scratchpad Virtual Research Environment (http://scratchpads.eu/) is a flexible system for people to create their own research networks supporting natural history science. Here we describe Version 2 of the system characterised by the move to Drupal 7 as the Scratchpad core development framework and timed to coincide with the fifth year of the project’s operation in late January 2012. The development of Scratchpad 2 reflects a combination of technical enhancements that make the project more sustainable, combined with new features intended to make the system more functional and easier to use. A roadmap outlining strategic plans for development of the Scratchpad project over the next two years concludes this article.

## Introduction

In recent years the value of data as a primary research output has been increasingly recognised (RIN 2011). New technology has made it possible to create, store and reuse datasets, either for new analysis or for combination with other data in order to answer different questions. Such data were typically made available as supplementary files published alongside their respective papers or submitted to data repositories that are linked back to the supporting publication. Either way, the act of data preservation happened close to the time of publication, and usually some considerable period after the dataset was initiated. This time lag acts as a major barrier to the development of public archives for research data.

At this crucial time when researchers would rather be dealing with the final development of their paper and moving on to new projects, they are asked to deal with the considerable challenge of formatting and depositing data, often using complex data standards that may be unfamiliar to the contributors. In these circumstances identifying the correct metadata to describe versions of these data is a major challenge, particularly since research practices increasingly involve large multi-contributor datasets that have developed and evolved over a considerable period of time (Smith 2009). Coupled with concerns about the risk of exposing data before the originators have fully exploited it, and the lack of standard norms for citing data, all but the most committed researchers are likely to be unmoved by calls to publish their data. As a result, data deposition is usually something of an afterthought for most researchers, with current efforts arguably driven by mandates from research funders and journal editors, rather than self-motivated individuals (Costello 2009).

A solution to this problem is to embed the process of data creation, archival and storage into a system that supports the research practices of the contributor community, a process made easier by the steady migration away from paper-based note taking and into direct electronic capture. This must support the data management needs of a project from its inception through to publication and store the entire data workflow, taking into account methodological steps that alter the data (such as equations and processing algorithms) throughout. With this as a goal the collection of accurate metadata about the lifecycle of these data can be captured, with the final data suitably structured for archiving. This is especially important to researchers that would rather not hand off control of their data to remote strangers. When the time comes to deposit data (at publication or the end of funding), the relevant information could easily be transferred to a different, public storage repository, or made more widely accessible within the system in which it was created, for public access.

A general class of systems that support this process are Virtual Research Environments (VRE). Their purpose is to help researchers to work collaboratively by managing the increasingly complex range of tasks involved in carrying out research on both small and large scales (Carusi and Reimer 2010). The concept of VREs is still evolving, but the term can be understood as a shorthand for the tools and technologies needed by researchers to do their research, interact with other researchers (who may come from different disciplines, institutions or countries) and to make use of resources and technical infrastructures available at local, national, and sometimes international scales. Critically, a VRE must incorporate the context in which those tools and technologies are used. As a result the detailed design of a VRE will depend on many factors including the research discipline and security requirements.

Scratchpads (http://scratchpads.eu/) are an example of a VRE framework that has been constructed to support the needs of specialists interested in natural history (Smith et al. 2009). The system allows people to create their own website that supports the particular needs of their research community by selecting a personalised choice of features, visual design, and constituent data. Within any one Scratchpad network, users self-assemble their data and activities, often around user-defined or imported vocabularies (including biological classifications). These vocabularies provide a mechanism for navigating and structuring content. They can also provide a quality control framework for standardising certain types of data. Each Scratchpad includes service layers that provide integration, analytical and publication functions that add considerable value to the user. The original Scratchpad architecture is described in Smith et al. (2009), which details the motivation for the project as well as the original technical framework that supports the system. Two full time developers lead the technical development of the platform, which is presently hosted on a single virtual server at the Natural History Museum, London. Additional developers contributing software modules used by the Scratchpads are based at several other institutions in the UK, continental Europe and the US. Development proceeds according to an agile model with the overall vision and direction managed by a wider group of stakeholders that are closely connected to the user community.

**Figure 1.**
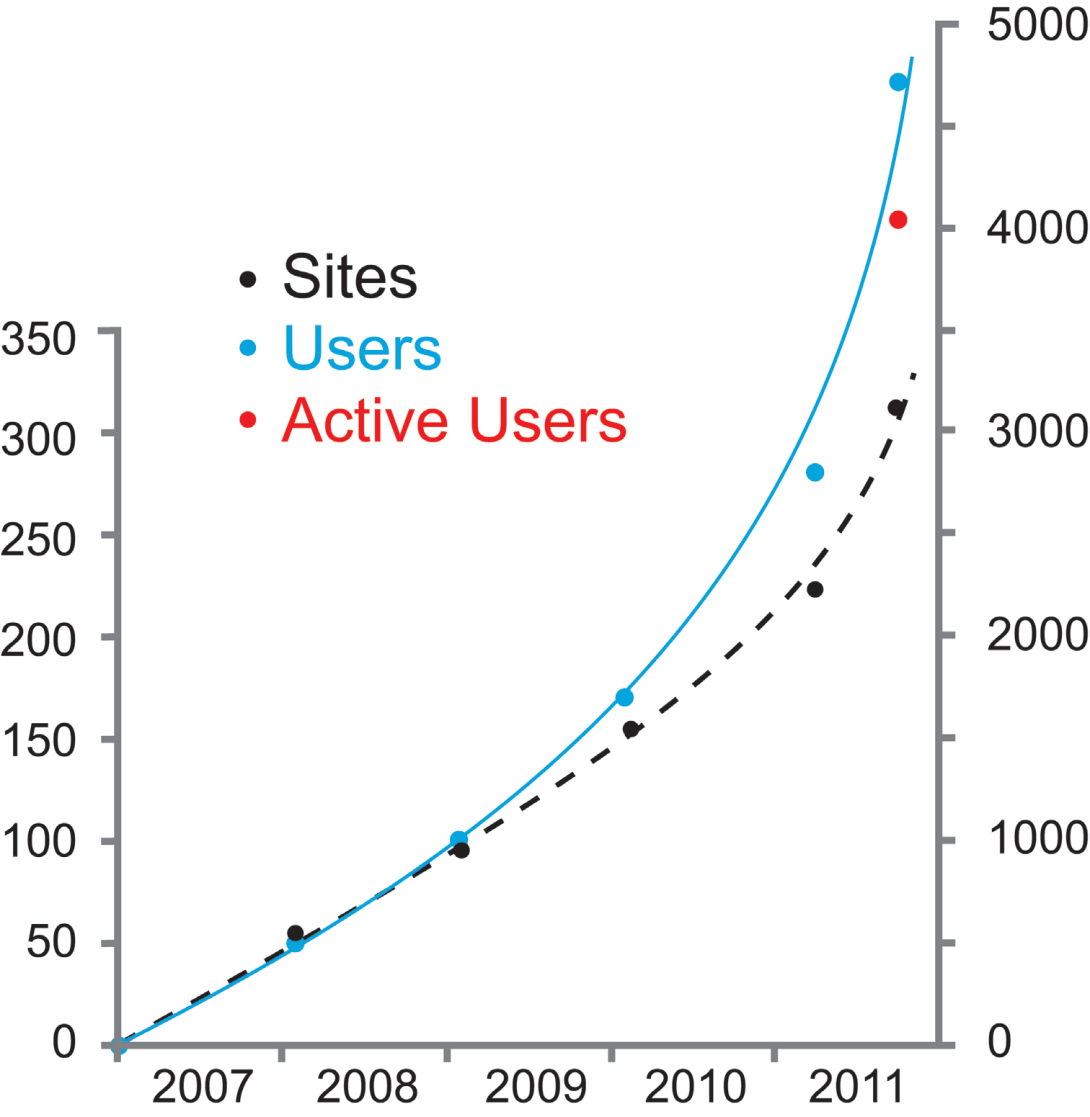
Scratchpad usage statistics from February 2007 to September 2011. The black dashed line represents the number of Scratchpad community sites (in hundreds) and the blue solid line represents the number of registered users (in thousands). As of September 2011 we have switched to recording the number of active users (currently 4424) since this figure provides a more accurate guide to usage.

In September 2011 there were over 300 Scratchpad community networks running on the Scratchpad platform (http://scratchpads.eu/scratchpads/stats). Thematically, these networks reflect the varied interests of natural historians, but can be broadly broken down into sites concerning specific groups of taxa, biogeographic regions or projects and societies. Networks range from 1 to 1,049 registered users (mean, 15, mode 1), and are composed of a mix of professional scientists and amateur naturalists. Just 17 Scratchpad networks have more than 50 contributors and almost half of all networks (129) have only one contributor. Contributor number is not necessarily indicative of quality or impact of a network, since two of the ten most visited Scratchpads have just two contributors each. Collectively the Scratchpad platform had over 4,400 registered and active users who have created 337,507 pages (nodes) of content between February 2007 and September 2011 (Figure 1). Scratchpad networks are free to all users. During January to September 2011 the Scratchpads received an average of 41,000 unique visitors per month across the platform.

February 2012 will mark the fifth anniversary of the Scratchpad project. It will also mark the planned release of a major new version of the software that incorporates many new features. This work is possible thanks to the EU FP7 funded ViBRANT project (http://vbrant.eu/), which is an e-Infrastructure initiative designed to support the development of virtual research communities. Additional support is provided by the NERC funded eMonocot project (http://e-monocot.org/). This paper provides a description of new features that will be released in Scratchpads 2, the motivation behind their development, and a roadmap for the future development of the Scratchpads over the next few years. As such it builds on the technical description of the Scratchpads provided in Smith et al. (2009) and does not duplicate descriptions there unless the concept or the functional component has changed substantially since originally being described.

## Implementation

### Development framework

Since their inception the Scratchpads have been developed using the Drupal (http://drupal.org/) Content Management System (CMS). Drupal offers a modular framework within which core functionalities can be readily extended through the development of new modules, or use of an extensive library of contributed modules. This approach means that the Scratchpads can make use of an extremely large community of contributing developers that provide core functionalities common to many web-based applications (e.g. user management), in addition to a smaller pool of distributed developers providing niche functionality that have general applications within the system (e.g. bibliographic management). This makes the Scratchpad project more sustainable as it allows funding to be focused on the development of functionality specific to the biodiversity sector that is of direct application to the Scratchpads.

The Scratchpads were initially released in Drupal version 5 as part of the EU funded European Distributed Institute of Taxonomy project (EDIT, http://www.e-taxonomy.eu/). At the end of 2008 the Scratchpads were upgraded to Drupal version 6, and new modules have been constantly developed or modified since. Version 2 of the Scratchpads has been developed using Drupal 7, which offers significant benefits over previous versions (see Table 1).

**Table 1.** List of new Scratchpad 2 features. A list of features that are either new or significantly improved in Scratchpad 2, with short mention of major benefits and the previous Scratchpad 1 method.

**New SP2 feature**	**Major Benefit**	**Previous SP1 method**
Single primary units (entities)	Reuse of code, better linking & better normalizing	Three primary units (nodes, users & taxonomy)
Editing via overlay module (opaque editing environment)	More space, more intuitive link between editing and viewing of content	Editing in central area of webpage
Workflows	Easier navigation of tasks that involve multiple independent actions	Complex actions required to pursue a single goal (e.g. set up a site or import data)
More intuitive user interface	Easier navigation, more efficient use of space	Sometimes confusing and cluttered user interface
Consistent theming	More consistent and user-friendly layout of sites	Large choice of themes, colour schemes and layout
Profiles (project specific SP templates)	Specific configuration settings, choice of modules and theme for a set of project sites	Only one SP template for all sites
Integrated point and area maps	Display of specimen data, regional distribution and GBIF data in one map	Separate point and distribution maps, separate map with GBIF data
Extension of publication module	support of wider range of datasets and manuscripts; wider range of journals	Only prototype publication module available
Import from Excel files, dynamic templates generated directly from SP	Generation of import files much easier, data validated against controlled vocabularies before import	Import via comma or tab delimited UTF-8 files, only few templates available
Integration of a HTTP accelerator	Improved overall performance of Scratchpad platform	Application server accessed directly
Use of native RDF (Resource Description Framework) [planned for later instance of SP2]	Display of data embedded as RDF within the HTML allows content to be machine readable without the need for dedicated services	Only limited services for external data harvesting (e.g. harvesting of taxon descriptions by EOL)

### Site management and distributed hosting

From April 2011 the Scratchpads adopted Ægir (Aegir, http://www.aegirproject.org/) as a site management tool. This provides a Drupal based hosting front end for the entire Scratchpad platform including all versions of the Scratchpads and Scratchpad training sites. Our configuration for Aegir allows sign up data to be automatically fed into the new site creation process, such that new sites can be set up in just a few clicks. To register for a new Scratchpad a user just has to complete a validated sign up form and the new Scratchpad is created automatically without any intervention by the Scratchpad development team. Backup and site upgrades are also managed by Aegir. Aegir also allows the Scratchpad team to deploy different Scratchpad profiles that have been developed to support sites with a subset of the full Scratchpad functionality (see below).

User feedback surveys have indicated a strong desire by more experienced users to host their own Scratchpads on a local server that is under their control. Until recently all production Scratchpads (i.e. publicly accessible sites in long term use) have been hosted at the NHM London. Attempts to host Scratchpads at other institutions have occurred, but none of these have gone beyond an experimental stage. As part of the ViBRANT project, technical development of the Scratchpads has enabled the existing NHM sites to be mirrored at the Botanic Garden and Botanical Museum Berlin (BGBM). In 2012, it will be possible to install new production sites on the BGBM server and we anticipate additional servers to come online in the near future. By distributing the hosting of the Scratchpads we hope to reduce the overall load on the NHM server that increasingly often reaches its performance limit when there are a high number of concurrent users. These distributed sites will also be centrally managed through the Scratchpad Aegir site (http://get.scratchpads.eu/).

### Scratchpad project profiles

Interest in the Scratchpad project is more and more coming from project based initiatives in addition to individuals. The data-gathering needs of these projects usually map to a subset of the full functionality offered by the Scratchpads, but may require a high level of customisation and standardisation in order to support the efforts of a particular initiative. Using the same site model as the Scratchpads, these initiatives allow communities of users to construct data according to templates specific to an initiative, and often have particular branding requirements that identify that the sites are part of a common effort. As part of Scratchpad 2 we can now support this functionality through the development of dedicated Scratchpad *profiles*. These profiles contain configuration settings, a list of modules to install, alternative themes and additional site setup settings that are specific to a particular initiative. Modifications to the Aegir site management have enabled us to deploy project specific profiles in the same way as regular Scratchpads. At present the only project to make use of this functionality is eMonocot (http://e-monocot.org/), which aims to create a global online resource for monocot plants by collating data provided by taxonomists working through dedicated eMonocot Scratchpads. There are, however, several potential applications for Scratchpad site profiles, including the GBIF (Global Biodiversity Information Facility) nodes portal toolkit, which is intended to be a mechanism for member countries to establish a web presence and view a subset of relevant species observation records from GBIF (http://www.gbif.org/). Another potential application of Scratchpad profiles are “LifeDesks” (http://www.lifedesks.org/). These are currently deployed in Drupal 6 by the Encyclopedia of Life (EOL) project (http://eol.org/) and are functionally very similar to the Scratchpads.

### Code management

The Scratchpad project is Open Source and released under a GPL version 2 license. Originally the codebase was managed through a dedicated SVN repository. This was converted to a Git repository (https://git.scratchpads.eu/) in February 2011 to stay with the same system used by Drupal itself and to improve the development environment.

Within the repository there are two Scratchpad code branches. One (master) is used for development and contains the latest version of the code. This is inevitably unstable being the development environment, and it is less thoroughly tested than the second (stable) code branch. Code is released to the stable branch on an intermittent cycle, after it has been subjected to user acceptance testing by a trusted subsection of the Scratchpad user community.

### Data services

A common criticism of version 1 of the Scratchpads was that each site was a data silo that lacked two-way connectivity to the wider landscape of biodiversity informatics initiatives (Page 2009). This criticism is partially justified. Scratchpad taxon pages provide significant inbound connectivity via the API’s of a diverse collection of biodiversity projects and within the Scratchpads an increasing number of users are providing data via outbound connectivity to third party projects such as the EOL. Also users have long had the capability to create their own dynamic CSV or XML feeds on any data type present within the Scratchpads. Despite these functions, usage of the outbound connectivity from the Scratchpads is comparatively low. This problem will be addressed within Scratchpad 2 by applying data services to all content by default, and more prominently advertising the presence of these functions.

Within Scratchpad 2 we will supply DwCA format, along with the appropriate extensions, for the majority of content. In some cases DwCA format is inappropriate or unsupported by external systems and services that are currently in use. For example, EOL species pages presently harvest Scratchpad content in a version of the Species Profile Model XML format. Likewise, the Scratchpad character project exports data in a variety of well-known formats for which there is no obvious DwCA extension. In these cases the present output formats (Structured Descriptive Data, Lucid format and Nexus format) will be maintained to keep interoperability with a wide array of third party applications.

DwCA files will be created at regular intervals for each site, as a background task, because building the archives is a comparatively slow process. We plan to drive this off the underlying database so that the archives dynamically reflect modifications to the structure of the site. Thus as new fields are added to the entity type, which define the appropriate DwCA extension field, their content will be dynamically mapped to the DwCA file when it is next created.

### Consistent theming

For each current Scratchpad site the maintaining user (i.e. the site coordinator with administrative privileges) could choose between any of the default themes that came with Drupal 6. Some maintainers also selected themes from those on Drupal.org and requested that they be uploaded to their sites. Depending on the options that came with each theme, users could select to have menu-bars on the left, right or both sides of the page, customise the arrangement of content within these menu-bars, and alter the colour scheme. As a consequence some Scratchpad maintainers employed idiosyncratic layouts and colour schemes that did not make their site visually appealing to the widest possible audience.

As part of Scratchpad 2 this problem is addressed by the development of a new dedicated Scratchpad theme that provides less layout and colour scheme flexibility. This new theme will enforce compatibility with the Web Accessibility Initiative (WAI) Double-A standards (http://www.w3.org/WAI/). The theme will nevertheless offer a significant degree of customisation while allowing the Scratchpad development team to exploit a higher degree of layout standardisation. The goal is to present content in a more consistent and user-friendly way across all the sites. Dedicated themes will be developed for separate site profiles as these come on stream, allowing collections of sites to conform to the brands of commissioning initiatives. Note that this design decision will present certain challenges for existing sites, some of which may struggle to conform to the restrictions imposed by the new site theme.

### Site administration

Users administrating version 1 of the Scratchpads found this a complex process because many administration functions are not intuitive, hard to physically find on the administrative interface, and when selected, their effect was often not immediately apparent. As part of the Scratchpad 2 release the administration back end has been completely redesigned with a new dedicated administration theme. This provides more intuitive grouping for the administration functions and makes the link between the cause and effect of each feature more obvious. For example, the options to configure menu-bar content are directly accessible from the menu-bar and altering these settings has an immediate visible effect. The administration functions also benefit from the full width display of the *overlay* module that provides a visual indication that the user is performing an administrative action.

### Taxon pages

Scratchpad taxon pages provide a mechanism for users to dynamically construct and curate pages of information about any taxon selected from the site’s biological taxonomy. These pages use taxonomic names as a search term to integrate tagged content in a Scratchpad with third party content external to the site. This third party content draws upon a variety of external data sources (e.g. *Biodiversity Heritage Library*, *flickr*, *GBIF* and *NCBI Genbank*), which have suitable APIs that support this type of integration.

The original implementation of taxon pages in Scratchpads version 1 suffered from a number of problems. These relate to the scientific accuracy of the third party content, the content selection interface, and the visual presentation of content, which may be poorly displayed and hard to organise for certain types of data. In consequence, many Scratchpad communities do not use the taxon page feature, or turn off the majority of third party content because the burden of curating these pages outweighs their perceived benefit. As part of Scratchpads 2 the taxon pages have been significantly re-engineered to address these issues, in part by making much greater use of EOL species page content. This is a close match to Scratchpad taxon page data. EOL provides a rich API that allows third party projects to access this information. To this end Scratchpads version 2 will use EOL as the primary provider for third party taxon page content. In addition we will work with EOL to support the rating and verification of source material through the API, such that registered Scratchpad users will be able to feed back to EOL content ratings and validate the status of content. EOL species page content will be integrated with existing Scratchpad taxon page content with the corresponding source clearly identified. A filter will allow Scratchpad users to choose whether to display just their Scratchpad Content, Scratchpad and trusted EOL content, or Scratchpad and all EOL content. As in Scratchpads version 1, an on-demand citation can be generated for any taxon page that creates a permanent archived version of the page and a citation as well as a permanent URL for that page.

### Mapping

Scratchpads version 1 supports three types of maps:

1) Point locality maps using the Google Maps API and the *gmaps* module, which are constructed dynamically from any content type containing geolocation data. Point locality maps are primarily used with Scratchpad specimen records but can also be applied to other appropriate content such as users.

2) The recording of taxon presence / absence distributions conforming to the TDWG level 4 geographical scheme. This is enabled by the *country maps* module.

3) Third party distribution maps dynamically obtained from GBIF via their API.

At present these maps are independent from each other and in consequence it is possible for a user to display a species page showing three, potentially conflicting, distribution maps for the same taxon. As part of Scratchpad 2 we will integrate these maps so that point information, and regional distributions can be displayed together. This will be implemented through an improved *Google Maps* module that incorporates version 3 of the Google Maps API. Feeds of georeferenced data from multiple sources (e.g. GBIF and FLICKR) can be displayed as points on a map, in addition to areas corresponding to TDWG level 4. As part of ongoing development work we plan to make these externally supplied map points and their metadata locally editable, such that individual records can be hidden, and point metadata edited locally within the Scratchpad.

### Dynamic content templates and data import / export

Import mechanisms within Scratchpads version 1 operate on delimited text files for any content type (e.g. tab or comma delimited files, usually generated by users from spreadsheets). In addition, specific import mechanisms are provided for a limited number of additional data types including biological taxonomies. As part of the Scratchpad 2 development, data can now be imported directly into a site using an Excel template, omitting the need to convert the file into a delimited text file format. The template is dynamically constructed from the Scratchpad, ensuring that it reflects any underlying changes to the entity type, in much the same way that the DwCA and extension files do. Furthermore, this Excel template can incorporate validation directly from the user’s Scratchpad. For example, a user may wish to import specimen records that directly link to a biological taxonomy that has already been embedded in the user’s site. The template incorporates this taxonomy as a separate worksheet connected to the column containing the specimen records taxon name so that records are validated before the import. The goal is to improve the user experience and reduce the number of errors that occur during data imports. The templates also contain embedded help text to guide users through the process of preparing their data. Technically this is made possible by the Drupal* feeds* module and the PHPExcel library.

### Scratchpad workflows

Research on Scratchpads (Smith et al. 2010) and the Drupal CMS (http://drupalusability.org/) suggest that navigating tasks involving multiple independent actions (e.g. importing a biological taxonomy, or administrative tasks like adding new users) is the single greatest usability issue within the system. The problem has a significant effect on user retention because many users become frustrated when performing tasks that are infrequently required but have a profound impact on their site. Likewise, the need to perform complex actions, especially in the early stages of setting up a site, has been demonstrated to be one of the biggest barriers to entry for many new users (Smith et al. 2010).

In an attempt to address these issues the *form-flow* module has been developed by the Scratchpad team. This supports the construction of workflows, which are a mechanism to link together complex actions that would otherwise require the use of multiple forms, editing environments and menu selections in pursuit of a single goal. Form-flow allows the Scratchpad development team to integrate multiple-step forms into a single “flow”. When a user complets the series of forms, they are collectively submitted as part of a single action. Error checks and validation are performed at every step, and users can navigate backwards and forwards between the component forms without loss of data. Within Scratchpad 2 form-flows exist for site setup functions; adding users and associated permissions; importing content including biological taxonomies; creating new entity types; publishing and exposing data through a service; and creating customised views of data. The entry point to these form-flows will replace the existing start point for these tasks, although maintainers will still have independent access to the underlying elements of a form-flow. In addition, maintainers can construct form-flows through the user interface.

### Matrix editing

The matrix editor addresses the problem of how to edit multiple records for any entity in an intuitive editing environment while making efficient use of space within a webpage. The matrix editor emulates spreadsheet functionality in a web browser. The module (http://drupal.org/project/slickgrid) makes use of the jQuery slickgrid plugin (https://github.com/mleibman/SlickGrid) and defines a view-style in which all data can be handled within an editable grid. Features of the *slickgrid* module include grouping fields (to link logically connected fields); support for collapsible taxonomy fields (tree structures, such as those representing biological classifications); tabs (to organise columns under tabs); deletion of multiple entities (e.g. rows) via the grid; multiple undo (to revert previous changes) and many more functions (see the module description at http://drupal.org/project/slickgrid for full details).

### Character projects

The *character project* module is built on top of the *slickgrid* module and defines specialised plugins dedicated to describing the molecular and morphological phenotype of organisms. This enables users to manage complex collections of morphometric, text and DNA character states that are optionally controlled via selection of a limited number of predefined states. The data editor allows datasets to be entered, changed, and has numerous features for manipulating rows, columns, and blocks of data, and for recoding data. It supports the import and export of SDD (Structured Descriptive Data), Nexus and Lucid data files, and is intended to provide the framework for a more integrated suite of analytical and visualisation tools that will support the production of identification keys, phylogenetic trees and natural language descriptions of taxa. The c*haracter project* module also makes use of the advanced entity relationships possible in Drupal 7. These allow metadata to be recorded about the connection between one or more entities. For example, within the character project this provides a common method for states to be annotated with images, text and bibliographic references present within a Scratchpad database.

### Publication module

**Figure 2.**
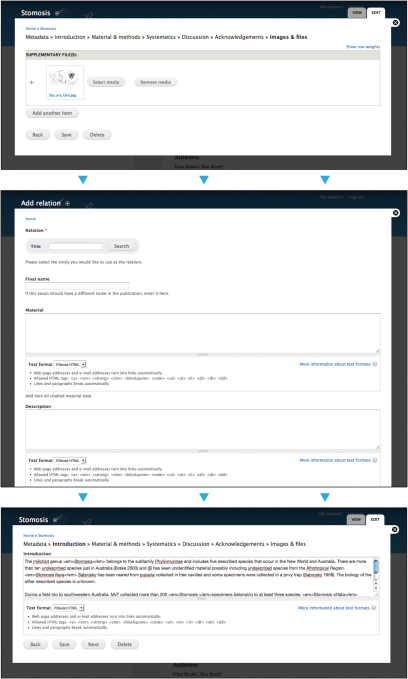
Screenshots of the Scratchpad 2 publication module showing an example workflow. Top, the section writing tool showing material and methods section; middle, the relationship selector that allows a taxon and additional materials to be associated with a section of the publication; and bottom, supplementary files such as illustrations, photos or graphs can be added to complete the publication.

A major long-term goal for the Scratchpads is to support users throughout the complete lifecycle of their data, from the inception of a project, through to its publication. As part of Scratchpads version 1 a prototype module was built that supported this functionality. This was outlined by Blagoderov et al. (2010) who described a method to publish nomenclatural acts via Scratchpads that are formally registered in the printed journal *Zookeys*. The workflow supports the generation of manuscripts directly from the Scratchpad database and is extended in Scratchpad 2 to support the construction of a wider range of datasets and manuscripts for submission to several additional endpoints. Within the first release of Scratchpads 2 these endpoints are limited to the major Pensoft series of journals (*Zookeys*, *Phytokeys*, and *Mycokeys*), as well as the construction of *Red List Threat Assessments *(Figure 2). The latter enable Scratchpad users to document the risk of extinction to species within a political management unit according to precise criteria defined by the International Union for Conservation of Nature (IUCN). Other publishers can implement software to handle the XML output from a Scratchpad, delivered in the open TaxPub schema, and, once available, their journals can be added to the list of possible endpoints.

When the publisher’s API supports feedback mechanisms (such as comments received through peer review) the module will be further developed to automatically update the publication, with the goal of speeding up the process of editing the final document while maintaining an enduring link to the supporting data.

### Help and support services

The Scratchpads have employed a variety of mechanisms over the past five years to support users (see Brake et al., this volume for a full review). Despite these advances, providing adequate support to a rapidly growing number of users remains an ongoing challenge. This is a particular problem with agile software development methods that can result in the rapid development of user interfaces, which occasionally require users to relearn tasks they previously performed by another method. To address this a help desk was formally established with the appointment of a dedicated user support manager in January 2010. The help desk deals with all the emails, issues, calls and meetings relating to user support. In September 2010 a custom-built issues tracker (http://dev.scratchpads.eu/project/issues) was developed that provided a mechanism for administrative users to report bugs, access support and make feature requests directly from their own Scratchpad, without the need to log into a separate system.

As part of the ViBRANT programme, basic and advanced training courses were organised to support and extend the Scratchpad userbase. These one-day courses are free of charge, paid for under the ViBRANT grant, and are intended to help current and prospective Scratchpad owners develop their site building skills, learn best practices and gain a better understanding of what Scratchpads can do for their research communities. A distance learning package has also been developed for those unable to attend a training course in person. Further, the help system has been extensively re-developed to become context-sensitive which helps novice users to control their Scratchpads. Throughout 2011 an extensive survey has been undertaken to further identify how the needs of users can be better supported. Full details of this are available in Brake et al. (2011) published in this volume.

## Discussion

Prioritisation of these development activities for Scratchpad 2 have been conducted in close coordination with the user community, via feedback mechanisms that have been solicited and collated by a team within the ViBRANT project. This work has provided insight into the technical and social challenges faced by contributors when using the Scratchpads. Research into the motivation behind user engagement with the Scratchpads (Smith et al. 2010), has also led to the development of technical innovations designed to sustain engagement and expand the existing userbase. With these results in mind the development of Scratchpads 2 reflects a combination of backend enhancements intended to make the technical maintenance of the project more sustainable at a larger scale, coupled with new frontend features intended to make the Scratchpad system more functional and easier to use.

Based on the amount of time involved in the development of Scratchpads 2, the transition to Drupal 7 has proven harder than originally anticipated. We estimate that 13 person-months of developer time have been spent on transition of Scratchpads from Drupal 6 to Drupal 7. This compares to just 3 person-months on the Drupal 5 to Drupal 6 transition. However, the comparison of effort is not equal because subsequent developments to the Drupal 6 version of the Scratchpads have made the system much more complex and feature rich. For example, the initial Drupal 6 version of the Scratchpads contained fewer than half the number of Scratchpad specific modules than the Scratchpads contained just prior to the Drupal 7 redevelopment. In addition, the Drupal 7 transition has resulted in a complete redevelopment of the Scratchpad architecture.

Further complications to the development of Scratchpad 2 involve the transition to *entities* and *relations*, which are a defining feature of the Drupal 7 core architecture. These features were very poorly documented on Drupal 7’s release in early January 2011. Drupal is an Open Source project and therefore dependent on volunteer contributions to upgrade; consequently, it has taken a very considerable period of time for Drupal developers to re-write their modules to take advantage of these functions in contributed modules relevant to the Scratchpads.

Despite these challenges, we expect the Drupal 7 transition to provide a much more sustainable platform on which to innovate and provide continued developments. Priority areas for development after the Scratchpad 2 release include:

– The production of a central registry for all the Scratchpad sites providing metadata on every entity type in each Scratchpad. This will also log user contributions, providing a mechanism to quantify activity that can be converted into a single contributor metric. In addition the registry will display statistics about non-contributing visitors. Registry functionality will replace the existing statistics pages at http://scratchpads.eu/scratchpads/stats and will be driven by enhancements to the *scratchpadify* module.

– Improved integration of polytomous keys and semi-automated construction of natural language taxonomic descriptions. These will be dynamically driven by the *character project* module that supports the documentation of taxon phenotypes, rather than statically creating keys from one-time exports, as is the case with the current Scratchpads.

– Integrated Single Sign On (SSO) across the Scratchpads, enabling users to access multiple Scratchpads with an existing login (such as a user’s Google, Facebook, or Yahoo ID) rather than creating a new user login for each Scratchpad.

– Integration of Digital Object Identifiers (DOIs) for select content within Scratchpads. At present a number of communities are using the Scratchpads as a system for distributing specialist journal articles such as the *European Mosquito Bulletin* (http://e-m-b.org/) and Phasmid Studies (http://phasmid-study-group.org/content/Phasmid-Studies). Others are archiving datasets that have a persistent and lasting value to the wider community (e.g. the comprehensive citations of Milichiid flies at http://milichiidae.info/content/citation). In an effort to formalise these outputs so that they are independently registered and citable we will be exploring the assignment of CrossRef DOIs to journal articles, and DataCite DOIs to datasets. Implementation of this function raises a challenge with respect to distributing the hosting of the Scratchpads and the maintenance of URL links. Nevertheless, this is an essential step for this output to become more readily accepted as formal scholarly content.

– The Scratchpad home site (http://scratchpads.eu) will be rebuilt with an emphasis on dynamically showcasing content from current Scratchpads, rather than emphasising the software.

– There will be greater integration of external analytical and vocabulary services into the Scratchpads. These will be driven by new developments from the ViBRANT programme, and include access to the catalogue of services available to the Oxford Batch Operation Engine (https://oboe.oerc.ox.ac.uk/) and developments to the GBIF controlled vocabularies server (http://vocabularies.gbif.org/).

– The Scratchpad training materials will be redeveloped with both botanical and zoological examples and will include support for training non-maintainer contributors from within a single taxonomic community (presently these materials focus on maintainers from multiple communities). As part of this redevelopment we will incorporate more standardised approaches to the training content that clarify the goal of a training task, alongside the prerequisites for its delivery, rather than just providing a set of step-by-step instructions and screenshots.

An ongoing issue with the Scratchpads and all e-infrastructure projects is finding an enduring model that secures their financial sustainability. In practice a mixed approach will be necessary for the Scratchpads, which relies on a combination of core support from institutions with a vested interest in the project, in addition to funds from external grant awarding bodies to drive innovation and new developments. As part of this mixed model we will be looking at opportunities to raise modest amounts of revenue from existing Scratchpad communities. This will take the form of value-added services such as priority technical support, maintenance of a persistent resolver for DOI identifiers on content, and data parsing services to facilitate the rapid construction of site.

## Conclusions

We describe Scratchpads 2, a Virtual Research Environment supporting scholarly collaboration, communication and data publication in biodiversity science. This represents a significant upgrade on the existing Scratchpad infrastructure. The original system has been in operation for five years demonstrating a clear demand for a structure of this type. The changes described here considerably expand the technical stability and functional capabilities of the system allowing the infrastructure to continue to grow at a sustainable cost. These changes include new tools to manage the distribution and hosting of sites, data services on all content, more consistent theming, new taxon pages, integrated mapping, dynamic content templates, workflows, new data editing environments, a new publication module and improved user-support functions. The guiding principle used during the development of Scratchpads 2 has been to construct a scholarly communication system that closely resembles and is intertwined with the scholarly pursuit of natural history, rather than being its after-thought or annex. We would be the first to admit that Scratchpad 2 does not fully deliver this aspiration, but we believe that it lays sustainable groundwork towards this goal.

## Availability and requirements

**Project name:** Scratchpads

**Project home page:** http://www.scratchpads.eu/

**Operating system(s): **Platform independent (Web application)

**Programming language:** PHP

**Other requirements:** none

**License:** Web application is freely accessible for all users. Source code is available under GNU General Public License version 2.

**Content:** remain the property of the contributors published under Creative Commons by-sa-nc licence.

**Restrictions to use:** none

## Authors’ contributions

VSS designs and leads the project, and coordinates the biological, sociological and technical insight that defines the Scratchpad program of work. SDR leads all aspects of the technical development, writing and integrating the package of software and providing the system administration. SDR also manages the additional technical developers including BS who has designed and constructed many of the complex editing and publication interfaces present within Scratchpad 2. EB has developed the profile functionality within the Scratchpads, in particular the eMonocot profile and associated training materials. EB alongside IB, LL, DR and VB provide selected testing and user support. IB leads the user support work and development of the training materials. DR realised, with VSS, the original Scratchpad implementation under the EU project EDIT (contract number 018340) and project manages the Scratchpads as part of the ViBRANT project, tests modules and develops selected functionality on selected sites. VSS wrote the manuscript. Other authors provided editorial comments and approved the final draft.
